# The clinical and functional relevance of microparticles induced by activated protein C treatment in sepsis

**DOI:** 10.1186/cc10356

**Published:** 2011-08-11

**Authors:** Margarita Pérez-Casal, Victoria Thompson, Colin Downey, Ingeborg Welters, Duncan Wyncoll, Jecko Thachil, Cheng Hock Toh

**Affiliations:** 1 Roald Dahl Haemostasis and Thrombosis Centre, Royal Liverpool University Hospital, Prescot Street, Liverpool, L7 8XP, UK; 2 National Institute of Health Research Biomedical Research Centre, Royal Liverpool University Hospital, Prescot Street, Liverpool, L7 8XP, UK; 3 Department of Clinical Infection Microbiology and Immunology, Institute of Infection and Global Health, University of Liverpool, 4th Floor University Clinical Departments, Duncan Building, Daulby Street, Liverpool, L693GA, UK; 4 Critical Care Unit, Guy's and St. Thomas' NHS Foundation Trust, Lambeth Palace Road, St Thomas's Hospital, London, SE1 7EH, UK

## Abstract

**Introduction:**

Activated protein C (APC) induces release of microparticles (MP) from primary physiological cells, which are found in patients undergoing treatment with recombinant human APC (rhAPC) for severe sepsis. We hypothesised that APC on these circulating MPs activate endothelial protease-activated receptor 1 (PAR1) to induce anti-apoptotic and anti-inflammatory properties that can improve patient outcome.

**Methods:**

This was an experimental study on clinical samples in an intensive care setting, and included patients with severe sepsis who fulfilled criteria for treatment with rhAPC. The number of CD13+ MPs from the patients were analysed to determine their origin. They were also quantified for endothelial protein C receptor (EPCR) and APC expression. Clinical relevance of these MPs were ascertained by comparing survival between the group receiving rhAPC (*n *= 25) and a control group of untreated patients (*n *= 25). MPs were also incubated with endothelial cells to analyse apoptotic gene expression, cytoprotection and anti-inflammatory effects.

**Results:**

rhAPC treatment induced a significant increase in circulating MP-associated EPCR by flow cytometry (*P *< 0.05) and by quantitative ELISA (*P *< 0.005). APC expression also showed significant increases (*P *< 0.05). Numerically, CD13+ MPs were higher in rhAPC-treated survivors versus non-survivors. However, the number of non-survivors was low and this was not significantly different. APC on MPs was demonstrated to induce anti-apoptotic and endothelial barrier effects through the activation of endothelial PAR1.

**Conclusions:**

rhAPC treatment in patients with sepsis significantly increases circulating EPCR + MPs. These MPs were noted to express APC, which has specific anti-apoptotic and anti-inflammatory effects, with a non-significant correlative trend towards survival. This suggests that MPs could disseminate APC function and activate endothelial PAR1 at distal vascular sites.

## Introduction

The presence of circulating microparticles (MPs) in septic patients is well recognised [1,2] and is inducible by thrombin [3], cytokines [4], lipopolysaccharide (LPS) [5] and collagen [6]. Derived from cell membrane shedding as a result of activation or apoptosis, circulating MPs constitute a marker of vascular and systemic disease [7]. Rearrangement of membrane phospholipids during MP release can result in increased phosphatidylserine availability with procoagulant activity. In patients with myocardial infarction and diabetes mellitus, elevated MP levels correlate with increased thromboembolic risk [8,9]. However, their functional role in the pathophysiology of sepsis remains unclear.

Elevated circulating MPs do not cause thrombosis in healthy individuals, principally due to the protective effects of the natural anticoagulant, activated protein C (APC) [10]. APC is an anticoagulant [11] with anti-inflammatory and anti-apoptotic properties [12]. These beneficial effects may be explained by its binding to the endothelial protein C receptor (EPCR) with cleavage of endothelial protease activated receptor 1 (PAR1) [13]. Although, the relative *in vivo *usefulness of these effects are not yet known, recombinant human APC (rhAPC) is currently used to treat patients with sepsis [14]. Its current use remains controversial because of reports of severe bleeding complications during rhAPC treatment [15] and a second phase 3 trial is ongoing (PROWESS Shock) [16].

We have previously demonstrated that APC can generate MPs *in vitro *from EPCR-expressing cells, which retain anticoagulant and PAR1-dependent anti-inflammatory properties [17]. *In vivo *demonstration of these APC-MP in septic patients during rhAPC infusion [18] led us to hypothesize that such circulating MPs may retain their anti-inflammatory, and cytoprotective properties in these patients. An increased number of these MPs would thus translate into clinical benefits for the patient with severe sepsis.

## Materials and methods

### Circulating MP-associated EPCR

Circulating MPs were from patients diagnosed with severe sepsis (American College of Chest Physicians criteria) [19], who also fulfilled the National Institute of Clinical Excellence (England and Wales) criteria [20] for rhAPC (Drotrecogin alfa (activated)) (Xigris^®^, Eli Lilly, Houten, Netherlands) treatment. The type of organisms isolated included pneumococcus, enterococcus, enterobacter, coagulase-negative staphylococcus and staphylococcus aureus. Patients who received a 96-hour continuous infusion of rhAPC (24 μg/kg/hr) were contrasted with an equal number of patients who were eligible but not treated because of concerns over bleeding risks. These concerns included gastrointestinal bleeding within six weeks (2), platelets < 30 × 10^9^/L (6), internal bleeding (3), intracranial pathology (1), chronic severe liver disease (4), recent major surgery (7) and trauma (2). None of these patients were on heparin prophylaxis because of bleeding concerns and all rhAPC-treated patients received low molecular weight heparin prophylaxis before and after but not during rhAPC infusion. The study protocol was approved by the Local Research Ethics Committee and the Research and Development departments of the Royal Liverpool University Hospital and Guy's and St. Thomas' Hospitals. Informed consent was obtained from patients or when patients were unable to consent, assent was sought from their next-of-kin for enrolment into the study and publication of results. Written consent was also obtained from six healthy normal donors who provided blood samples for MP isolation. A copy of the written consent is available for review by the Editor-in-Chief of this journal. Blood samples were collected into 0.105 M trisodium citrate with and without 0.1 M benzamidine. From each patient, this was six blood samples. In the rhAPC-treated group, this was before rhAPC initiation and then at 24, 48, 72 and 96 (during rhAPC infusion) and 120 hours (post-rhAPC treatment). In the non-rhAPC treated group, corresponding time points for blood sampling were also used.

CD13 (aminopeptidase N) is a trans-membrane protease present in endothelial cells and recognised as a marker for MPs arising from these cells. EPCR, the cellular receptor for protein C and activated protein C, is primarily localized on endothelial membrane and has been demonstrated to be expressed on endothelial MPs. Using fluorescence-activated cell sorting (FACS) [17], CD13 and EPCR were enumerated from a MP-specific gate based on size and calculated in relation to 3 μm latex (polystyrene) beads. MPs were isolated by initial centrifugation of plasma at 5,000 g for 10 minutes to remove cells, followed by further centrifugation of the supernatent at 18,000 g for 30 minutes twice at 4°C. EPCR and APC on MPs were measured by ELISA, as previously described [17]. In brief, MPs were captured by anti-EPCR-RCR2 and detected using chromogen S2366 or anti-EPCR RCR49-biotin for quantifying APC and EPCR, respectively. All assays were performed blinded to knowledge of rhAPC treatment or its timing. For experiments assessing functional effects, platelet-derived MPs were removed as these are not induced by APC [17] by first labelling with CD41 (platelet glycoprotein IIb)-phycoerythrin (PE) for 15 minutes and mixed with anti-PE magnetic beads (Miltenyi Biotech, Bisley, Surrey, UK) for 20 minutes on ice. Wash-through was collected for CD41-negative MPs with negativity confirmed by FACS. Clinical data were collected, including baseline characteristics and outcome at 28 days.

### Functional assays

In addition to the clinical studies of MP number, functional assays were also performed to assess the effect of rhAPC-expressing MPs on endothelial cell function. Details of reagents and methods used to determine endothelial gene expression are available in Additional file 1. As previously reported [21,22], a 1.5-or greater fold-change in hybridization intensity was used to denote significant regulatory change in gene expression. The effect of MPs on endothelial permeability was analysed in a dual chamber system using Evans Blue-labelled bovine serum albumin (BSA) [23]. In brief, EAhy926 cells (endothelial cell line) were plated on 6.5 mm diameter Transwell polycarbonate membranes of 3 μm pore size (Costar, Corning, NY, USA). Upper and lower chambers were filled with 100 μL and 500 μL of growth medium, respectively. Cells were grown for two days to obtain monolayers that were "sub-confluent". As described by Feistritzer [23], the sub-confluent model enables any improvement in endothelial barrier integrity to be detected; that is, reduction in leakiness by the agonist. Blocking antibodies and inhibitors were added 30 minutes before APC or MP. Cells were exposed to APC or MP for three hours and permeability assessed afterwards by adding 0.67 mg/ml Evans Blue in medium containing 4% BSA to the top chamber. After 10 minutes, an aliquot was taken from the lower chamber and diluted 1:2 in medium for measurement at 650 nm. Further studies on the influence of zymogen protein C on APC binding to MPs [24] was based on the binding assay of Regan *et al. *[25] (Supplementary Materials and methods).

### Statistical analysis

**Figure 1:**
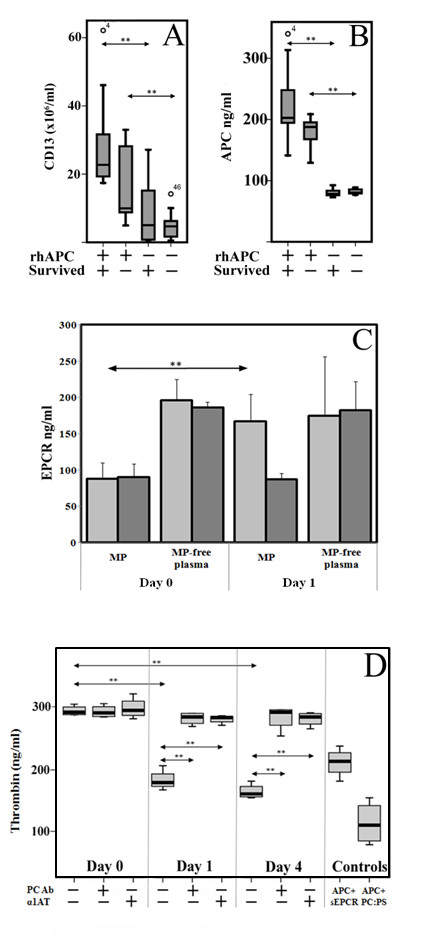
**The clinical correlation of activated protein C-induced MPs with survival, EPCR expression and anticoagulant properties**. Figure 1 shows the effect of rhAPC treatment on **(A) **CD13+ MPs, and **(B) **APC levels on MPs. MPs from 50 septic patients obtained on Day 1 (25 treated with rhAPC and 25 matching untreated non-rhAPC patients) were analysed by flow cytometry for CD13 positive MPs/ml of plasma. MPs were stained with PE IgG control or CD13 antibodies. Levels of APC on the MPs were quantified using S2366 by ELISA on MPs isolated from rhAPC-treated or non-treated patients. Survival at 28 days is denoted by + (*n *= 16 for both treated and untreated groups) and death by - (*n *= 9 for both groups). ** indicates significance of *P *≤0.016 by Mann-Whitney U test with Bonferroni correction. **(C) **Levels of EPCR expression were measured by ELISA on MPs isolated from plasma and the residual MP-free plasma from six rhAPC treated patients (light bars) and six matching untreated patients (dark bars) obtained on Day 0 and Day 1 (of rhAPC treatment or controlled time-point in non-rhAPC group). **P < 0.05 indicates significance difference between pre- vs. during-rhAPC as assessed by a Mann-Whitney U test. **(D) **APC activity on MP from six patients on Days 1 and 4 was estimated by the ability to generate thrombin after a prior Factor Va incubation step. The MPs were also incubated with Protein C antibody (PCAb) and α_1_-antitrypsin (α_1_AT) for assessing APC specificity, with APC plus phospholipid vesicles (APC + PCPS) as positive control and APC plus soluble endothelial protein C receptor (sEPCR) as negative control. The mean values ± SD are from duplicate samples in three experiments from each patient. *P < 0.05 and **P < 0.005 indicates significant comparisons between Day 0 vs. Day 1, Day 0 vs. Day 4 in the absence and presence of PC antagonists.

Data were expressed as the mean and standard deviation (SD) of at least four independent experiments. For MP quantification in Figure 1A, B, data were expressed as the median with interquartile ranges (IQR). In data that were not normally distributed, Mann-Whitney U tests with Bonferroni correction were used.

## Results

### APC treatment in patients with sepsis induces the release of EPCR-expressing MPs

In initial experiments on six rhAPC treated patients, FACS demonstrated a significant increase in CD13 and EPCR expressing MPs (*P *< 0.05) within 24 hours of the treatment (Table 1). Due to sample volume limitations from the critically ill patients and concordant information from CD13 and EPCR expression in the available cases, further analyses (50 patients) utilised only CD13 positivity as a marker of EPCR-expressing MPs. Table 2 highlights the characteristics of these groups along with the APACHE II scores, sepsis aetiology, vasopressor treatment, DIC scores, ITU stay and survival data, which were comparable. A trend towards lower platelet counts was, however, noted in the non-rhAPC treated group.

**Table 1:** CD13 and EPCR positive (+) microparticles in response to recombinant human activated protein C (rhAPC)

	CD13+ (x 10^6^/ml)		EPCR+ (x 10^6^/ml)	
**Patient No**.	Pre-rhAPC	During rhAPC	Pre-rhAPC	During rhAPC
1	4.88	8.21	2.8	5.16
2	7.77	41.33	5.8	27.55
3	6.27	24.38	3.37	16.53
4	8.56	12.42	6.62	11.42
5	1.92	71.30	1.06	54.41
6	21.83	35.92	13.1	33.04
Median	7.02	30.15*	4.59	22.04*

During rhAPC indicates day 1 of treatment. *= p ≤ 0.025 comparing pre- vs. during treatment effects using a Mann-Whitney U test with Bonferroni correction.

**Table 2:** Patient characteristics

	rhAPC treated (*n *= 25)	Non-rhAPC controls (*n *= 25)
Age, years*	70 (51 to 82)	61 (52 to 71)
**Men/Women, n**	13/12	14/11
PT, seconds*	17.6 (15.0 to 21.1)	18.1 (15.8 to 22.4)
PLT, × 10^9^/l*	128 (55 to 218)	102 (25 to 298)
WBC, × 10^9^/l*	19 (8 to 25.9)	19.3 (9.4 to 33)
APACHE II, score*	29 (25 to 35)	28 (24 to 34)
**Aetiology of sepsis, n**	Pneumonia 16 Intra-abdominal infection 9	Pneumonia 13 Intra-abdominal infection 12
**Septic shock**	8	9
Noradrenaline, (μg/kg/minute)*	0.4 (0.2 to 0.7)	0.4 (0.2 to 0.9)
**Overt DIC^+^**	8	11
Total days on ITU, n*	8 (6 to 18)	18 (10 to 52)
**Survival at 28 days**	16	16

Numbers (n) with * indicate median values (minimum to maximum).

^+ ^as defined by ISTH criteria [44].

Figure 1A shows that the number of CD13+ MPs was higher (*P *= 0.1) in patients that underwent rhAPC infusion and survived (22.7 × 10^6^/ml, IQR 18.5 to 31.5) compared to rhAPC treated non-survivors (10 × 10^6^/ml, IQR 8.2 to 28.9). The level of CD13+ MPs in the non-rhAPC treated survivor group were not significantly different statistically from non-rhAPC treated non-survivor patients (*P *= 0.19).

The amount of APC on the generated MPs was measured by ELISA (Figure 1B). The levels were significantly higher during rhAPC treatment compared with individual, pre-treatment controls and unrelated, non-rhAPC treated patient controls (Figure 1B). In all the 25 rhAPC-treated patients, the level of APC detected in MPs was between 120 and 350 ng/ml. Daily monitoring showed no significant variation in levels. Levels were higher from the rhAPC survivors (202.6 ng/ml, IQR 194.5 to 251.7) compared to the rhAPC treated non-survivors (187.5 ng/ml, IQR 160.2 to 198.4) (*P *= 0.04). These data were compared with our *in vitro *studies which showed that the proportion of rhAPC that is bound to MPs following APC stimulation is approximately 6 to 10% (Additional file 2). To assess if APC on MPs could be due to passive binding to already circulating MPs, rhAPC was added to plasma samples from non-rhAPC-treated septic patients for 24 hours. The results showed less than 20 ng/ml APC in these cases (data not shown), indicating that findings during rhAPC treatment reflect APC bound to circulating MP-associated EPCR. Specific EPCR ELISA indicated a significant increase of EPCR levels on MPs during rhAPC treatment (160 ng/ml) in comparison to pre-treatment levels (80 ng/ml) (Figure 1C). This was also demonstrated to be not because of passive binding of sEPCR to MPs, as sEPCR levels in MP-free plasma were unchanged from pre- to rhAPC-treatment samples.

### MP-associated EPCR from APC-treated patients express anticoagulant activity

**Figure 2:**
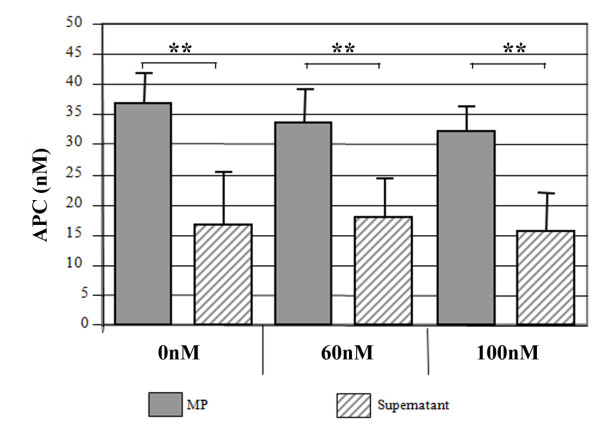
**Effect of protein C (PC) on circulating activated protein C (APC)-induced microparticles (MPs)**. Levels of APC quantified by chromogenic S2366 on MPs isolated from patients (Day 2 of rhAPC treatment) compared to separated supernatant following 30 minutes of incubation with 0, 60 and 100 nM PC. Data represent mean values from four patients assayed in duplicate. **indicates *P *< 0.005 statistical significance comparing MPs to corresponding supernatant.

The functional aspects of MP released during rhAPC treatment were next analysed. Figure 1D shows a substantial loss in thrombin generation by these MPs compared with pre-treatment stage. Mean thrombin generation fell from approximately 294 ng/ml to 184 ng/ml during rhAPC infusion. This was reversible by PC blockade or the APC inhibitor, a_1_AT. By contrast, patients not treated with rhAPC did not express this anticoagulant property (data not shown). This indicates that a functionally active circulating form of APC exists on MP-EPCR during clinical treatment with rhAPC. APC levels on MPs were observed to be similar in the absence or presence of PC. This indicates that APC on patients' MPs (*n *= 4) are not easily displaceable as levels in the separated supernatant remained unchanged even at very high PC concentrations (Figure 2).

### Circulating MP-associated EPCR affects APC-specific anti-apoptotic gene expression

**Figure 3:**
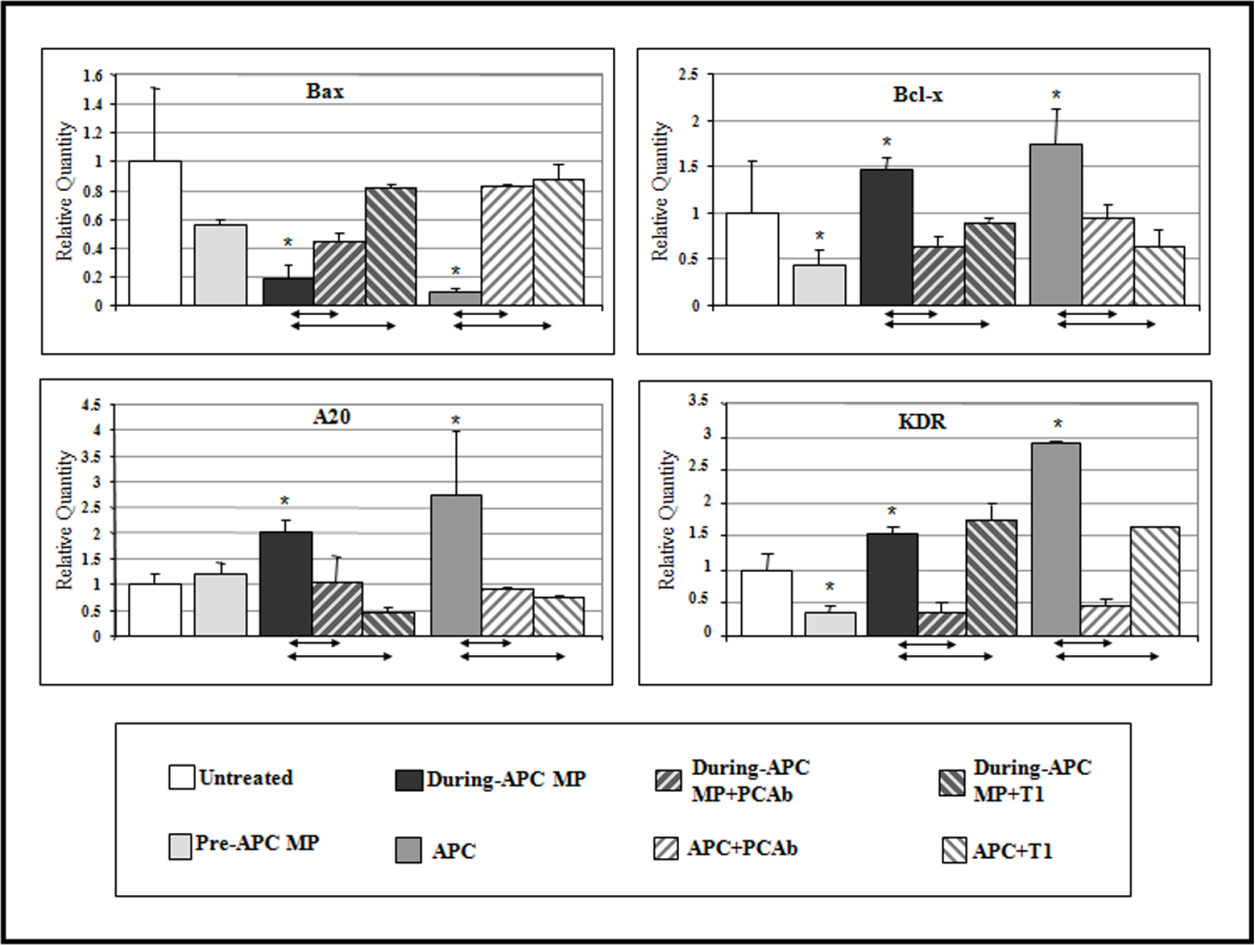
**Effect on the differential expression of selected genes by circulating microparticles bearing activated protein C**. Human umbilical vein endothelial cells were stimulated with microparticles (MPs) isolated from septic patients before (Day 0) and during (Day 1) recombinant human activated protein C (rhAPC) infusion compared to an equivalent concentration of free APC. Bax, Bcl-x, A20 and KDR genes were analysed by quantitative polymerase chain reaction and expressed relative to untreated cells, which were set at 1. Data from six patients are presented as mean ± SD. *P < 0.05 denotes significant comparisons to untreated, with significant influences by protein C (PC) blockade or antagonist T1 denoted at base of chart.

Since APC on MP-EPCR in plasma retain anticoagulant activity, we postulated that they could modulate genes involved in apoptosis and inflammation. As the majority of circulating MPs are platelet-derived and platelets do not express EPCR [26], these were depleted to obtain an EPCR-enriched MP pool. The efficiency was 95 to 98% depletion and the vast majority of these CD41-depleted MPs were CD13 and EPCR positive (data not shown). When these MPs (approximately 0.15 × 10^6^) were tested on gene arrays, those isolated during rhAPC treatment induced the expression of anti-apoptotic genes including A20, Bcl-x and the vascular endothelial growth factor (VEGF) receptor 2/kinase insert domain-containing receptor (KDR) (Figure 3). In most cases, PC blockade reversed these specific expressions (Additional file 3). Exceptions to the expression of several anti-apoptotic genes were matrix metalloproteinase (MMP)-2, and MMP-1 to a lesser extent (PC blockade remained above the 1.5 cut-off level for significant regulatory change). Most transcript effects of APC were reversible by PAR1 antagonism with the exception of KDR. This could be because of PAR1-independent APC effects or insufficient PAR1 inhibition under these conditions.

### APC on circulating microparticles prevents apoptosis via PAR1 activation

**Figure 4:**
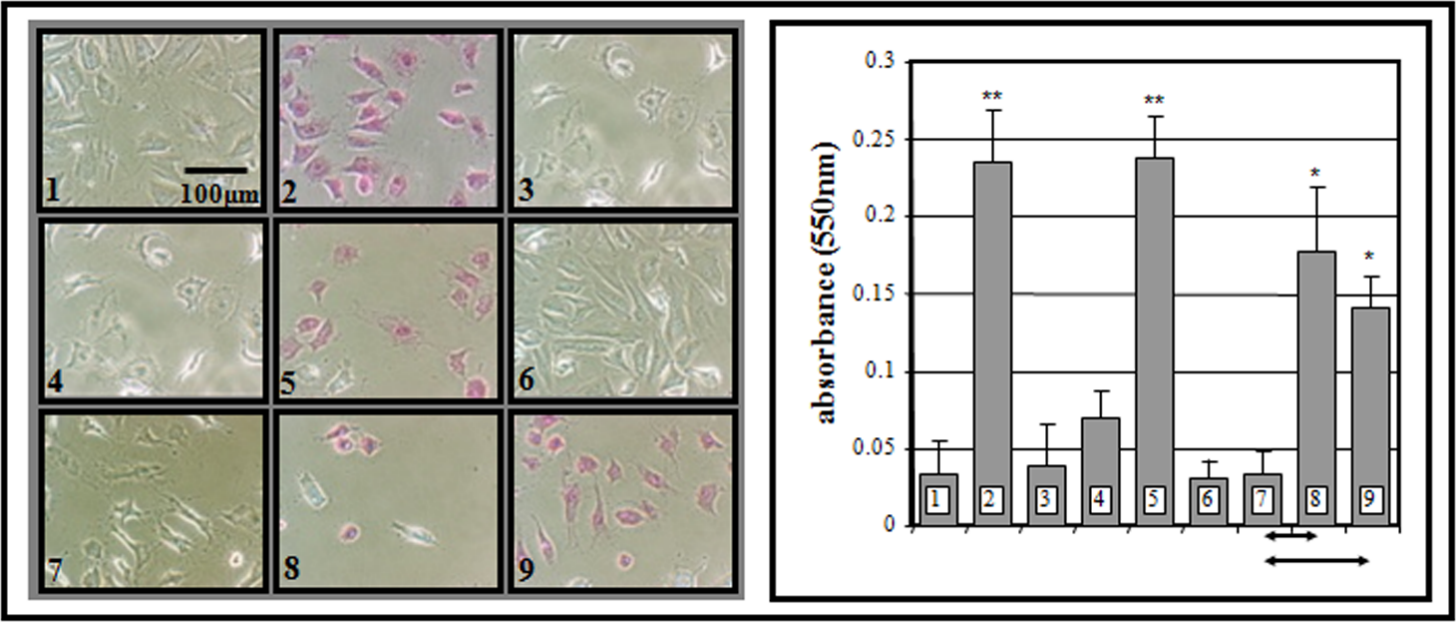
**Effect of circulating microparticle-bound activated protein C (MP-APC) on an endothelial model of apoptosis**. Light microscopy (X200 magnification) shows human umbilical vein endothelial cells: [1] untreated, and treated with [2] staurosporine (10 μM), [3] APC + staurosporine, [4] MPs from pre-rhAPC treatment (Day 0), [5] MPs from pre-rhAPC treatment + staurosporine, [6] MPs during rhAPC treatment (Day 4), [7] MPs during rhAPC treatment + staurosporine, [8] MPs during rhAPC treatment + Protein C (PC) Antibody (Ab) + staurosporine, and [9] MPs during rhAPC treatment + antagonist T1 + staurosporine. The bar chart shows corresponding release of up-taken dye with data from six separate experiments expressed as mean ± SD.*P < 0.05 and **P < 0.005 denote significant comparisons to untreated, with significant influences by PC blockade or T1 denoted at base of chart.

rhAPC-induced MPs could also inhibit apoptosis to a similar level as free-APC through a PAR-1 dependent mechanism (Figure 4). This cytoprotective property can be shown to be absent with PAR1 antagonism. Panel 8 (in Figure 4) shows that PC blockade did not, however, completely abrogate cytoprotective signals in all cells, suggesting that other cytoprotective mechanisms may exist in the context of rhAPC treatment. Another plausible explanation is that the MPs used were not 100% EPCR and APC positive. Nonetheless, these results indicate that circulating APC on MP-EPCR from rhAPC-treated patients have anti-apoptotic properties that are comparable to the free APC effect. Furthermore, this was not observed with MPs from non-rhAPC treated samples.

### Barrier-protective effect of circulating APC-MP in a subconfluent endothelial model is APC and PAR1 dependent

**Figure 5:**
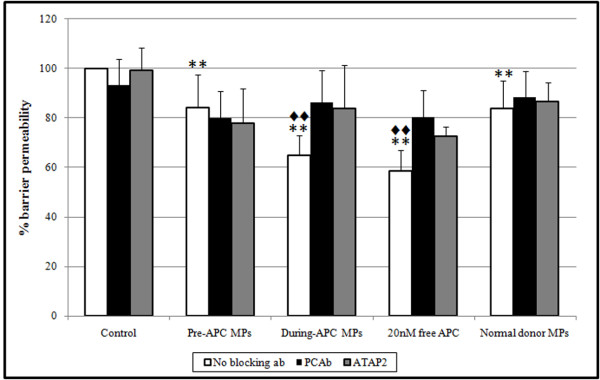
**Effect of free and circulating microparticle-bound activated protein C on endothelial permeability**. Endothelial cells seeded onto Transwell plates were incubated with 20 nmol/L free- **activated protein C (**APC) or with 1.4 × 10^5 ^circulating microparticles (MP), for three hours and permeability was assessed using Evans-Blue-Bovine serum albumin. MP were obtained from Day 3 of rhAPC treatment. Specificity of APC and protease activated receptor 1 (PAR1) was investigated by inclusion of protein C (PCAb) antibody (10 μg/mL) or PAR1 specific antibody (ATAP2 at 20 μg/mL) for 30 minutes before incubation with APC or MPs. All experiments were performed as duplicates; *n *= 3. **P < 0.006 denote statistical significance in relation to untreated baseline; ♦♦ *P *< 0.006 denote significance between APC or, microparticle-associated APC (MP-APC) and blockade as assessed by a Mann-Whitney U test with Bonferroni correction.

To further investigate a role for APC-MP on inflammation, the effect of free or MP-associated APC on endothelial permeability was examined. Using MPs from healthy donors as controls, we compared circulating APC-MP from three patients before and during rhAPC infusion. The same number of circulating MPs from healthy individuals or patients were applied; that is, 1.4 × 10^5 ^MPs per well. Figure 5 shows that circulating MPs from the rhAPC treatment period reduced permeability by approximately 35 to 40%, similar to free APC. This protective effect was again APC and PAR1 dependent. Notably, MPs taken from the patients before rhAPC treatment or those taken from healthy individuals had a minor protective effect that did not reach statistical significance. This was also neither APC- nor PAR1-dependent.

## Discussion

Our work is the first to demonstrate that APC can induce the generation and release of MPs *in vitro *and *in vivo*. A key finding from this study is that circulating MPs from patients during rhAPC treatment for severe sepsis possess APC-specific functional effects. The interaction between these MPs with endothelial cells can induce changes in gene expression, which translate into inhibiting apoptosis and reducing endothelial permeability. As these effects require PAR1 activation at the endothelial surface by APC in an EPCR-bound conformation [18], these results provide functional confirmation to earlier microscopy evidence of the assembled EPCR-APC complex on *in vivo*-derived MPs. Our findings also indicate that APC binding to EPCR within circulating MPs is stable and not displaceable by non-activated PC.

Whilst circulating MPs in sepsis and inflammatory conditions have been widely described, data on their function are limited. The majority highlight pro-inflammatory [27,28], pro-apoptotic [29] and pro-coagulant [30,31] potential of circulating MPs. Whilst increased MP levels in disease states are generally associated with adverse outcomes [32-34], Soriano reported that endothelial MP levels were higher in survivors of severe sepsis than in non-survivors [35]. Our findings would support this with rhAPC treatment significantly increasing endothelial-derived CD13+, EPCR+ MPs with a clinical correlative trend towards improved outcome. Expression of the functional EPCR-APC complex may be relevant to this trend in survival benefit as such MPs could bypass the requirement for cellular co-localisation of EPCR and PAR1 to induce APC anti-inflammatory and cytoprotective signaling [18]. As inflamed cell surfaces are EPCR deficient [36], MPs could be a vehicle for APC to act at a distance on PAR1 in other cells. This would reduce endothelial apoptosis and permeability in distal vascular sites, as supported by increases in anti-apoptotic A20 and Bcl-x with concomitantly reduced pro-apoptotic Bax. This would also reduce endothelial permeability as KDR up-regulation has barrier protective functions [18]. Our findings that APC-induced MPs are anticoagulant active suggest that these MPs might contribute to the bleeding risks associated with rhAPC treatment. No evidence of increased bleeding was, however, observed in the rhAPC-treated patient group.

Since thrombin can also cleave PAR1 to cause endothelial barrier disruption, the question as to how PAR1 mediates opposing effects has been debated and further clarified. Recently, APC-PAR1-dependent protection has been demonstrated *in vivo *in reversing inflammation-induced vascular leakage [37]. Such work in physiological systems, and also work in our own group [18], suggest that a key molecular differential is the APC-induced interaction between EPCR and sphingosine 1-phosphate that is important to cytoskeleton stability [38]. EPCR is co-localised with PAR1 in lipid rafts and APC facilitates their coupling to channel Gα_i_-PAR1 protective signalling [38]. Gα_i_-PAR1 recruitment also modifies the inner leaflet of plasma membranes and this may be mechanistically linked to MP formation [39]. With EPCR located within these glycosphingolipid-rich microdomains (rafts) and caveolae, this provides a likely pathway for calcium-regulated kinases and phosphatases, guanine nucleotide exchange factors and mitogen-activated protein kinase cassettes to form and release MP-EPCR [40]. As to thrombomodulin (TM) expression on APC-induced MPs, our investigations do not show a consistent presence (data not shown). This is not unexpected given that the cellular distribution of TM is different from EPCR [41] and MP generation by APC is EPCR-dependent but TM-independent.

A limitation of this study is in the numbers of rhAPC treated patients, especially with the relatively few deaths in the rhAPC treated group. To optimise the validity of our findings, we were careful to control for the rhAPC treated group with a group of patients who were equally eligible for rhAPC treatment but who were not treated due to clinician concern of bleeding risks. The platelet count in this group was significantly lower (Table 2) but should not have affected our MP findings or conclusions as platelets do not express EPCR. In addition, we have both inter- and intra-patient comparisons with sampling before and during rhAPC treatment. Through this and the comprehensive characterisation of both prognostic and functional aspects of circulating MPs, this study has taken a basic observational discovery along the translational pathway towards possible clinical relevance. It is important to note that our findings are limited to APC-induced MPs, which to our knowledge is not influenced by heparin or drugs that might have an endothelial effect. This is because the APC effect is specifically cell receptor-mediated, that is, via EPCR and PAR1. A limitation to clinical translation is that the data may not be applicable to the most seriously septic patients whose mortality is much higher than in the study patients.

Our findings are the first to highlight that there is an additional circulatory form of EPCR in human plasma, that is, MP-EPCR, which is different from soluble EPCR. Soluble EPCR is the cleaved, truncated form of EPCR (45kD) that cannot facilitate APC proteolytic anticoagulant activity [42]. Another novel finding is that this provides the first evidence in humans that the APC-PAR1 pathway is physiologically relevant because MP-EPCR release is dependent on PAR1 activation by APC [17]. Whilst the relative importance of free versus MP-associated APC remains to be clarified, free APC levels can be variable during treatment in patients with sepsis [43]. This may be due to stability and clearance factors or that cell-bound APC is not measured. Conversely, our findings that MP-associated APC are stable both in measurable levels and activities would point to physiological and clinical relevance as bioactive effectors in rhAPC-treated patients. This conclusion comes with the caveat that their clinical relevance requires further exploration, especially in larger numbers of patients with septic shock and higher mortality.

## Conclusions

In conclusion, treatment of septic patients with APC can induce the release of MPs, which express EPCR. These MPs exhibit similar functional aspects to endothelium-associated EPCR by expressing anticoagulant activity, modulating APC-related anti-apoptotic gene expression and providing endothelial barrier protective effect through APC bound to them.

## Key messages

• rhAPC treatment in patients with sepsis significantly increases the number of circulating EPCR/CD13 expressing microparticles (MPs), with a clinical correlative trend towards improved outcome.

• These MPs express APC, which has specific anti-apoptotic and anti-inflammatory effects.

• This work suggests that MPs could disseminate APC function and activate endothelial PAR1 at distal vascular sites.

## Abbreviations

APC: activated protein C; APACHE II: Acute Physiology and Chronic Health Evaluation II; BSA: bovine serum albumin; DIC: disseminated intravascular coagulation; ELISA: enzyme-linked immunosorbant assay; EPCR: endolthelial protein C receptor; FACS: fluorescence-activated cell sorting; ITU: intensive treatment unit; IQR: interquartile range; KDR: kinase insert domain-containing receptor; LPS: lipopolysaccharide; MMP: matrix metalloproteinase; MPs: microparticles; PAR1: protease activated receptor; rhAPC: recombinant activated protein C; sEPCR: soluble endolthelial protein C receptor; TM: thrombomodulin; VEGF: vascular endothelial growth factor

## Competing interests

The authors declare that they have no competing interests.

## Authors' contributions

MPC participated in the study design and coordination, carried out the molecular genetic studies and functional assays, and helped to draft the manuscript. VT carried out the MP number experiments and functional assays and helped to draft the manuscript. CD carried out the MP number experiments. IW and DW contributed patient samples. JT helped to draft the manuscript. CHT conceived of the study and participated in its design and coordination and helped to draft the manuscript. All authors read and approved the final manuscript.

## Supplementary Material

Additional file 1 **Supplementary information**. Supplementary materials and methods, and figure legend [17,18,45-48]. Click here for file

Additional file 2 **Supplementary Figure S1. The proportion of free versus bound rhAPC on microparticles**. Comparison of results obtained from patient samples to *in vitro *derived MPs. Click here for file

Additional file 3 **Supplementary table S1. Genes modulated by APC (free or microparticle [MP]-bound from patients)**. Microarray results. Click here for file
