# Supplementary material for: The clinical and functional relevance of microparticles induced by activated protein C treatment in sepsis
Source: Crit Care. 2011 Aug 11;15(4):R195. doi: 10.1186/cc10356 (PMC3387637; doi:10.1186/cc10356)
Supplement: Additional file 1 — Supplementary information. Supplementary materials and methods, and figure legend [17,18,45-48]. [file cc10356-S1.DOCX]

## SUPPLEMENTARY SECTION

## Supplementary Materials and Methods

## Reagents

Human factors Va, Xa, prothrombin, α-thrombin, APC and protein C (PC) blocking antibody were from Haematological Technologies (Vermont, US), S2366, S2238 substrates from Chromogenix Laboratory, (Milano, Italy) and O-Phenylenediamine dihydrochloride (OPD) from Dako (Denmark). Anti-rat immunoglobulin G (IgG, whole molecule) labelled with peroxidase or FITC, isotype control rat IgG (whole molecule), latex beads for MP quantification and α_1_-Antitrypsin (α_1_AT) were from Sigma (Poole, UK). CD13-PE, CD41-PE, corresponding IgG class control and goat-anti-mouse-PE were from BD Biosciences (San Jose, CA). PAR1 antagonist peptide H-Met-Ser-Arg-Pro-Ala-Cys-Pro-Asn-Asp-Lys-Tyr-Glu-OH (T1) came from Bachem (St Helens, UK), blocking antibody ATAP2 from Santa Cruz Biotechnology (Santa Cruz, CA) and WEDE15 from Beckman-Coulter (Buckinghamshire, UK). EPCR antibodies RCR-2, RCR-49 and RCR-121 were kindly provided by Dr K Fukudome and used as previously described [17]. Soluble (s)EPCR was a gift from Dr. H. Nakatake (Chemo-Sero-Therapeutic Research Institute, Saga, Japan).

**Determination of Endothelial Gene Expression**

GEArray Q-Series Human Endothelial Cell Biology Gene Array (SuperArray, MD, US) containing 112 tetra-spots of sequence specific cDNA of different human genes, including 5 housekeeping genes and blank controls was utilised. ELISA first ascertained the amount of MP-bound APC to enable comparison with an equivalent concentration of free APC. APC concentration on patient-derived MPs was standardised at 17nM. As controls, both free and MP-bound APC were incubated with anti-PC (10µg/ml) 1h prior to incubation. With MPs isolated from pre or non-rhAPC treated patients where there were no significantly quantifiable APC levels, an equivalent number of CD13 positive MPs were used to standardise across time points. PAR1 blocking was by a 30 min pre-incubation with the PAR1 antagonist peptide T1 (50µM) or the blocking antibody ATAP2 (20µg/ml). T1 and anti-PC were also used on their own as controls for transcript levels. An isotype class control was used in antibody blocking experiments. Human umbilical vein endothelial cells (HUVEC) were prepared by the method of Jaffe *et al* [45] and used at passage 2 or 3. Confluent HUVEC were serum-starved and incubated in serum-free Iscove’s modified Dulbecco media with an equal amount of free or MP-bound APC for 4h at 37°C and 5%CO_2_. RNA was extracted with RNeasy Mini-kit (QIAGEN, Sussex, UK) and probes were made using the Ampolabeling-LPR kit (SuperArray, MD, US) for hybridization. Gene signal was detected by chemiluminiscence with a Fuji LAS-1000 digital imaging system (FUJIFILM, Bedford, UK) and analysed with the GEArray Expression Analysis Suite (http://geasuite.superarray.com). Gene expression was corrected for background and normalised against median value of each array. A 1.5 or greater fold-change in hybridization intensity between control and stimulated HUVECs signified regulatory change. All data was normalised to actin, compliant with Minimum Information about a Microarray (MIAME) [46] and submitted to Gene Expression Omnibus (GEO) database (accession number GSE5661), thereby allowing data to be accessible to anyone using the accession number. QT-PCR confirmation of array data was performed, as previously described [18].

**Assays for antigenic and functional APC**

To ascertain the proportion between free or bound APC on MPs, confluent EA.hy926 cells were incubated for 18 hours with 20 or 100nM of rhAPC and the media harvested after. MPs were isolated and APC levels in the media (free) and on the MPs (bound) were respectively determined by ELISA using a monoclonal antibody to APC (kindly provided by Dr CT Esmon, Oklahoma) [47]. Assessment of APC activity chromogenically was with S2366. Anticoagulant activity of APC on circulating MPs in inactivating factor (F.)Va was assayed, as previously described [17, 48] with specificity verified using the inhibiting activity of α_1_AT and also anti-PC (10µg/ml). Apoptosis was induced in confluent HUVEC using staurosporine (10µM) for 1h. Effect of free or MP-bound APC for 3h prior to staurosporine, was also examined. Apoptotic cells were detected with APOPercentage Apoptosis Assay (Biocolor, Newtonabbey, Northern Ireland), as previously described [18]. APC specificity was examined by pre-treatment with anti-PC (10μg/ml) or isotype control. For PAR1, T1 (50μM) was added before APC inclusion in free or MP form. Images were taken using an Olympus CK2 microscope with 10X objective lenses.

**APC displacement assay**

APC-induced MPs from patients (day 2 of rhAPC treatment) were isolated and quantified for APC content by chromogenic S2366, as previously described [17]. Equal concentrations of MPs, standardised by APC content, were incubated in HBS (20mmol/L HEPES, 0.15mol/L NaCl, pH 7.4) / 2.5mmol/L CaCl_2_/ 1µmol/L hirudin at varying concentrations of PC (0, 60, 100nmol/L) at RT. After 30 min, the sample was centrifuged at 18,000g for 20 min. Following separation from supernatant, MPs were washed in HBS/ 2.5mmol/L CaCl_2_. Supernatant and MPs were then respectively incubated with S2366 and the absorbance read in a Spectramax plate reader (Molecular Devices). Results were estimated from an APC standard curve.

**Supplementary Legend**

**Supplementary Figure 1. The proportion of free versus bound rhAPC on microparticles.** MPs were harvested following incubation of confluent EA.hy926 cells with rhAPC (20 or 100nM) after 18 hours. Free rhAPC levels in the media and bound rhAPC levels on MPs were determined by ELISA using a monoclonal antibody to APC. Data is expressed as the mean±SD of two replicates from four independent experiments.
