# Supplementary figures and images for: The clinical and functional relevance of microparticles induced by activated protein C treatment in sepsis
Source: Crit Care. 2011 Aug 11;15(4):R195. doi: 10.1186/cc10356 (PMC3387637; doi:10.1186/cc10356)

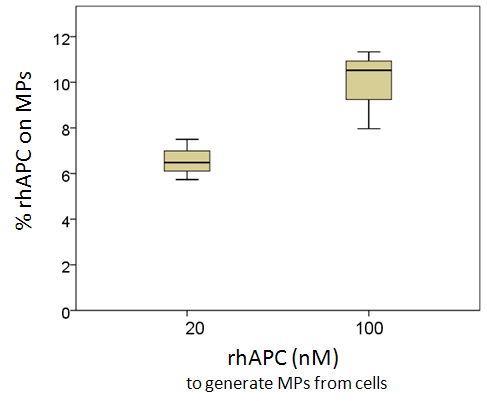

Supplement: Additional file 2 — Supplementary Figure S1. The proportion of free versus bound rhAPC on microparticles. Comparison of results obtained from patient samples to in vitro derived MPs. [file cc10356-S2.TIFF]
