# Supplementary material for: The clinical and functional relevance of microparticles induced by activated protein C treatment in sepsis
Source: Crit Care. 2011 Aug 11;15(4):R195. doi: 10.1186/cc10356 (PMC3387637; doi:10.1186/cc10356)
Supplement: Additional file 3 — Supplementary table S1. Genes modulated by APC (free or microparticle [MP]-bound from patients). Microarray results. [file cc10356-S3.DOCX]

**Supplementary table 1**. Genes modulated by APC (free or microparticle [MP]-bound from patients). Measurements are normalised and expressed as fold difference to untreated values.

| **Functional group/**  Gene name | | **APC (17nM)** | | | | | | ***In vivo* MP APC(17nM)** | | | | | **GeneBankNo** | |
| --- | --- | --- | --- | --- | --- | --- | --- | --- | --- | --- | --- | --- | --- | --- |
|  | | **APC** | | | **+**  **PCAb** | **+**  **T1** | +  **ATAP2** | ***Pre-rhAPC** | **During- rhAPC** | **During- rhAPC**  **+PCAb** | **During- rhAPC**  **+T1** | **During- rhAPC**  **+ATAP2** |  | |
| **Cell activation** | |  | | |  |  |  |  |  |  |  |  |  | |
| PAI-1 | | 3.5 | | | 1.006 | 1.12 | 0.26 | 0.87 | 1.54 | 1.12 | 1.07 | 0.26 | M16006 | |
| vWF | | 2.3 | | | 1.2 | 1.1 | 1.11 | 1.8 | 1.69 | 1.3 | 0.89 | 0.61 | NM_000552 | |
| **Pro-apoptosis** | |  | | |  |  |  |  |  |  |  |  |  | |
| Bax | | -1.8 | | | 0.43 | 0.6 | 0.5 | -1.6 | -5.5 | 0.59 | 0.89 | 1.05 | L22474 | |
| Caspase 6 | | -2.02 | | | 0.48 | 0.57 | 0.59 | -1.8 | -2.4 | 0.39 | 0.72 | 0.59 | U20537 | |
| CRADD | | -2.0 | | | 0.84 | 0.45 | 0.90 | 0.11 | -3.4 | 0.34 | 0.36 | 0.90 | NM_003805 | |
| **Anti-apoptosis** |  | |  | | |  |  |  |  |  |  |  |  | |
| Bcl-x | 3.1 | | 0.35 | | | 0.53 | 0.45 | 0.6 | 2.5 | 0.74 | 1.1 | 0.45 | Z23115 | |
| A20 | 3.3 | | 1.03 | | | 0.56 | 0.43 | 1.3 | 2.3 | 1.3 | 0.79 | 0.43 | NM_006290 | |
| TNFRSF10C | 1.6 | | 0.70 | | | 0.96 | 0.81 | 0.42 | 3.9 | 1.4 | 1.05 | 0.78 | AF016267 | |
| TNFRSF10D | 1.56 | | 1.27 | | | 1.03 | 1.22 | 1.06 | 3.02 | 1.23 | 0.65 | 0.42 | AF021232 | |
| **Angiogenesis** |  | |  | | |  |  |  |  |  |  |  |  | |
| FGF2 | -2.1 | | 0.49 | | | 0.89 | 0.51 | -1.5 | -1.9 | 0.88 | 1.13 | 1.15 | NM002006 | |
| MMP1 | 1.7 | | 1.2 | | | 0.74 | 0.93 | 1.3 | 3.06 | 1.52 | 0.37 | 0.39 | X05231 | |
| MMP2 | 1.69 | | 0.35 | | | 0.45 | 0.51 | 1.2 | 2.58 | 2.22 | 1.8 | 1.51 | J03210 | |
| KDR | 1.9 | | 0.69 | | | 2.6 | 1.10 | 0.85 | 1.5 | 0.98 | 2.05 | 1.62 | AF035121 | |
| **Adhesion** |  | | |  | |  |  |  |  |  |  |  | |  |
| ICAM2 | 3.6 | | | 0.43 | | 0.97 | 0.36 | 1.25 | 2.8 | 1.24 | 1.01 | 0.96 | | NM_000873 |
| Integrin aV | 1.6 | | | 1.2 | | 0.64 | 0.93 | 2.7 | 2.9 | 0.36 | 0.96 | 0.93 | | NM_002210 |
| Integrin beta3 | 2.4 | | | 0.91 | | 0.84 | 0.93 | 0.39 | 1.6 | 1.26 | 1.08 | 1.09 | | J02703 |
| VCAM1 | 2.01 | | | 0.64 | | 0.52 | 0.48 | 1.23 | 1.52 | 0.23 | 1.03 | 0.80 | | M30257 |

MP number standardized as by CD13+ (0.15x10^6^).
